# Alström syndrome: a cross-sectional and follow-up study of 127 patients in China, highlighting genetic variant spectrum and cardiac features

**DOI:** 10.1186/s13023-025-04139-8

**Published:** 2025-12-29

**Authors:** Yiguo Huang, Libo Wang, Qianwen Zhang, Shiyang Gao, Guoying Chang, Tingting Yu, Ru-en Yao, Yu Ding, Xiumin Wang

**Affiliations:** 1 Department of Endocrinology, Metabolism and Genetics, Shanghai Children’s Medical Center, Shanghai Jiao Tong University School of Medicine, Shanghai, 200127 China; 2 Department of Medical Genetics and Molecular Diagnostic Laboratory, Shanghai Children’s Medical Center, School of Medicine, Shanghai Jiao Tong University, Shanghai, 200127 China

**Keywords:** Alström syndrome, *ALMS1*, Cardiomyopathy, Echocardiography

## Abstract

**Background:**

Alström syndrome (ALMS) is a rare autosomal recessive multisystem disorder caused by biallelic pathogenic variants in the *ALMS1* gene, characterized by progressive cone-rod dystrophy, early-onset obesity, cardiomyopathy, and multiorgan dysfunction. Despite its clinical significance, comprehensive and population-based studies of ALMS remain limited worldwide, particularly in Asian populations.

**Methods:**

This cross-sectional and follow-up study enrolled 127 genetically confirmed Chinese ALMS patients (aged 1.0–35.1 years). Whole-exome sequencing (WES) identified *ALMS1* variants, and echocardiography evaluated cardiac phenotypes. Retrospective analysis tracked LVEF changes in 11 infantile cardiomyopathy cases.

**Results:**

A total of 132 distinct *ALMS1* variants (254 alleles) were identified, including 64 novel variants. Truncating mutations predominated (nonsense: 46.9%, frameshift: 45.3%), with recurrent variants c.10,825 C > T (7.9%), c.2090 C > A (5.5%), and c.10831_10832delAG (4.7%). Clinical manifestations included universal visual impairment (100%, 112/112), obesity (84.1%, 96/114), hearing loss (70.8%, 51/72), hepatic steatosis (66.7%, 40/60), and cardiac abnormalities (58.2%, 46/79). Echocardiographic analysis of 34 patients revealed exon 16 truncating variants were significantly associated with infantile-onset cardiomyopathy (72.7% vs. 30.4%, *p* < 0.05). In 11 infantile cardiomyopathy cases, LVEF improved post-treatment, yet progressive cardiac enlargement persisted.

**Conclusion:**

This study provides the most comprehensive clinical and genetic characterization of Alström syndrome in a Chinese cohort to date, identifying 64 novel *ALMS1* variants and expanding the known mutational spectrum of this rare disorder. The findings reveal the multisystem nature of ALMS, with frequent metabolic, hepatic, and cardiac involvement, and emphasize the importance of early recognition, multidisciplinary monitoring, and lifelong management. Together, these results establish an evidence-based foundation for improving genetic counseling, guiding precision diagnosis, and informing future mechanistic and therapeutic research on ALMS.

**Supplementary Information:**

The online version contains supplementary material available at 10.1186/s13023-025-04139-8.

## Introduction

Alström syndrome (ALMS; OMIM #203800) is a rare autosomal recessive disorder caused by biallelic mutations in the *ALMS1* gene, with an estimated global prevalence of approximately 1 in 1,000,000 individuals [1, 2]. To date, approximately 1,000 genetically confirmed cases have been reported worldwide [2]. The clinical presentation is highly heterogeneous, and does not follow a strictly sequential pattern. Although many patients present with early visual and auditory symptoms such as nystagmus or sensorineural hearing loss, others may first manifest severe infantile cardiomyopathy within the first months of life before sensory deficits become apparent [3]. Overall, ALMS is characterized by progressive multi-organ involvement, including visual and hearing impairment, childhood-onset obesity, insulin resistance, cardiomyopathy, and ultimately multiorgan fibrosis [1, 4].

The *ALMS1* gene (NM_015120.4), located on chromosome 2p13, consists of 23 exons and encodes a large protein of 4,169 amino acids primarily localized to the centrosome and basal body of cilia [5]. Mutations in *ALMS1* were historically classified as causing a “ciliopathy” due to the protein’s centrosomal localization and the associated defects in ciliary formation, maintenance, and signaling [6, 7]. However, recent evidence indicates that *ALMS1* also plays important non-ciliary roles in intracellular vesicular trafficking, cytoskeletal organization, and metabolic regulation, suggesting a broader cellular function beyond ciliary biology [8]. In *Alms1*-disrupted mouse models, loss of ALMS1 leads to abnormalities in endosomal recycling and vesicular transport [9], while further studies have demonstrated ALMS1 localization to non-centrosomal sites, including adipocytes, supporting its involvement in vesicle trafficking and insulin signaling [10, 11]. Collectively, loss-of-function mutations disrupt ciliary and non-ciliary signaling, including cross-talk between the TGF-β pathway and other related intracellular transport mechanisms, leading to structural and metabolic dysfunction [12, 13]. As of April 2023, the Human Gene Mutation Database (HGMD, https://www.hgmd.cf.ac.uk/ac/index.php) has documented 338 distinct *ALMS1* mutations, predominantly nonsense and frameshift variants that result in premature protein truncation. Mutational hotspots are mainly found in exons 8, 10, and 16 [2, 14]. However, significant gaps remain in the molecular genetic landscape of ALMS.

While prior investigations have advanced our knowledge of ALMS, existing evidence remains fragmented due to reliance on limited sample sizes (typically *n* < 50) and geographically restricted registries, predominantly from European cohorts such as Polish and Turkey populations [15–17]. Systematic studies in Asian populations, particularly among Chinese patients, remain scarce [18]. Furthermore, genotype-phenotype correlations, especially regarding the genetic basis and progression of cardiomyopathy, remain poorly understood due to limited patient numbers and a lack of longitudinal data.

This study recruited 127 genetically confirmed Chinese ALMS patients, establishing the largest clinical-genetic cohort reported in China to date. Whole-genome sequencing (WES) identified 132 distinct *ALMS1* variants, including 64 previously unreported pathogenic variants absent from major genomic databases or prior literature. Furthermore, this study conducted genotype-phenotype correlation analyses focusing on cardiac abnormalities and, for the first time, presented long-term echocardiographic follow-up data for patients with infantile-onset cardiomyopathy. Collectively, these findings yield valuable insights into the genetic and clinical characteristics of ALMS within the Chinese population, establishing an evidence-based framework to enhance genetic counseling, facilitate early diagnosis, and guide personalized therapeutic approaches.

## Materials and methods

### Participants

**Fig. 1:**
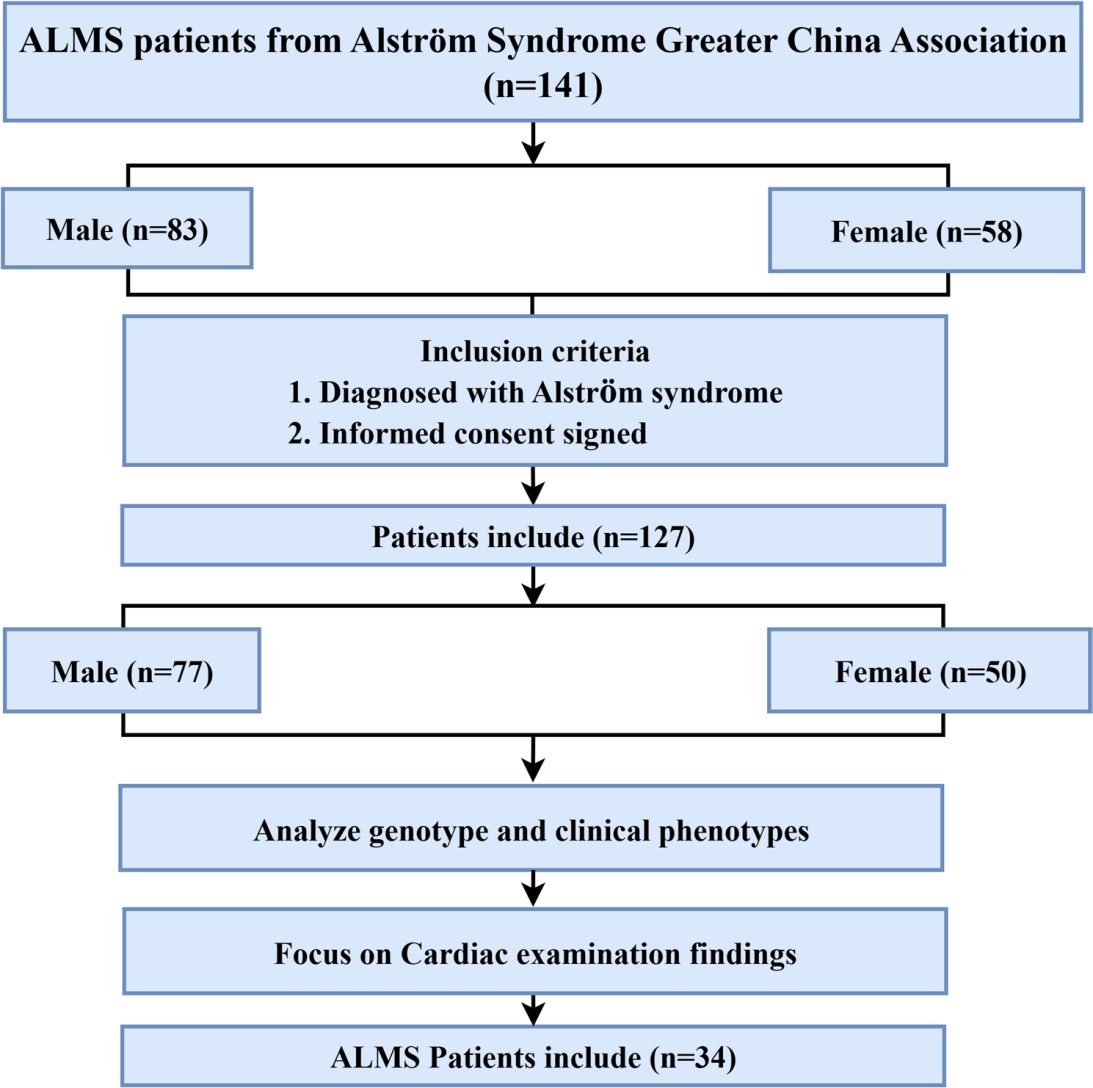
Study Flowchart. ALMS: Alström syndrome

This study was a single-center retrospective and follow-up observational study conducted at Shanghai Children’s Medical Center. The project was initiated in September 2019, integrating clinical, biochemical, and genetic data retrospectively collected from medical records traceable to 2010, with follow-up completed and data finalized in June 2025. A total of 127 individuals aged 1.0 to 35.1 years were recruited through the Alström Syndrome Greater China Association and the patient-centered ALMS group at Shanghai Children’s Medical Center (https://www.alstrom.cn/) **(**Fig. 1). The participants were diagnosed across multiple regions of China. Comprehensive phenotypic and genetic data were collected from all affected individuals, including demographic information, medical and personal histories, physical examination findings, and laboratory reports. All individuals had a genetically confirmed diagnosis of ALMS, established through molecular genetic testing and validated by experienced clinicians. The diagnostic criteria [19] included characteristic clinical manifestations, such as retinal dystrophy, obesity, diabetes mellitus, and cardiac abnormalities, supported by pathogenic (P) or likely pathogenic (LP) variants in *ALMS1*. Patients carrying variants of uncertain significance (VUS) were not included in the final analysis cohort. Written informed consent was obtained from all participants or their legal guardians prior to inclusion. The study protocol was approved by the Institutional Review Board of Shanghai Children’s Medical Center(Approval No. SCMCIRB-K2020060-1) and conducted in accordance with the Declaration of Helsinki. This study followed the Strengthening the Reporting of Observational Studies in Epidemiology (STROBE) guidelines (https://www.equator-network.org/reporting-guidelines/strobe/).

### Genetic sequencing

WES was performed in all participants to identify variants, which were subsequently confirmed by Sanger sequencing. Variant annotation was based on the RefSeq transcript NM_015120.4. In silico predictions of pathogenicity were performed using PolyPhen-2 (http://genetics.bwh.harvard.edu/pph2/), SIFT (http://sift.jcvi.org/), and MutationTaster (http://www.mutationtaster.org/ChrPos.html). The allele frequencies of all identified variants were below 0.1%. Variants were interpreted according to the American College of Medical Genetics and Genomics (ACMG) guidelines [20]. Only patients carrying P/LP *ALMS1* variants were included in the final cohort. Parental testing was performed whenever possible to confirm segregation. De novo variants were identified through trio analysis where both parents were available.

### Definition of insulin resistance

Insulin Resistance was defined using the homeostasis model assessment of insulin resistance (HOMA-IR) index: HOMA-IR = Fasting insulin (µU/mL) × Fasting glucose (mmol/L) / 22.5. A HOMA-IR value ≥ 3.16 was used as the diagnostic threshold for IR [21]. When fasting insulin or glucose data were unavailable, cases were classified based on documented clinical diagnosis of IR in the medical record. Non-fasting or incomplete data were excluded from IR analysis.

### Cardiac evaluations

Transthoracic echocardiography was performed using a Philips EPIQ 7 ultrasound system equipped with phased-array transducers (2–4 MHz for standard imaging and 3–8 MHz for tissue Doppler applications). All examinations were performed with patients in the left lateral decubitus position to optimize standard apical and parasternal imaging windows. Cardiac measurements were conducted in accordance with the guidelines of the American Society of Echocardiography [22]. For children aged under 5 years, echocardiographic images were obtained during quiet wakefulness or natural sleep with non-pharmacologic calming measures, such as parental comforting. If motion artifacts persisted, mild sedation was achieved with a single oral dose of short-acting chloral hydrate or intranasal dexmedetomidine, administered under continuous cardiorespiratory monitoring according to the institutional pediatric sedation protocol, ensuring patient safety and stable imaging conditions [23]. During peak diastolic opening, mitral inflow was recorded using pulsed-wave Doppler with the sample volume placed at the tips of the mitral valve leaflets. Tissue Doppler imaging (TDI) was performed by placing sample volumes at the septal and lateral mitral annulus to measure myocardial velocities.

### Statistical analysis

Categorical variables were presented as frequencies (%) and compared using the Pearson chi-square test or Fisher’s exact test, as appropriate. Continuous variables were expressed as mean ± standard deviation (SD) and compared using unpaired t-tests or non-parametric tests when applicable. Statistical analyses were performed using SPSS version 25.0 (Statistical Package for the Social Sciences Inc., Chicago, IL, United States). A two-sided P value of < 0.05 was considered statistically significant.

## Result

### Clinical features

A total of 127 patients (77 males and 50 females) from 117 non-consanguineous families were included. The mean age at disease onset was 0.71 years, while the mean age at diagnosis was 4.70 years (*P* < 0.001, Table 1). The predominant clinical manifestations included visual impairment (112/112, 100%), obesity (96/114, 84.1%), and hearing loss (51/72, 70.8%). Additionally, more than 50% of patients exhibited insulin resistance (47/80, 58.8%), cardiac abnormalities (46/79, 58.2%), and hepatic steatosis (40/60, 66.7%). Patients with insulin resistance were significantly older (mean 12.89 ± 5.60 years) than those without (7.61 ± 4.05 years, *p* < 0.001), supporting an age-dependent progression of metabolic impairment. Notably, a moderate positive correlation was observed between age of onset and diagnostic delay (*r* = 0.223, *P* < 0.05).

**Table 1:** The typical clinical features in 127 patients with ALMS

Features	Frequency (*n*/*N*, %)
Age at onset of initial symptoms (mean ± SD, y)	0.71 ± 0.66
Age at diagnosis (mean ± SD, y)	4.70 ± 4.43
Current age (mean ± SD, y)	9.84 ± 5.82
Gender (M/F)	77/50
Obesity (n/N, %)	96/114 (84.1)
Hearing impairment (n/N, %)	51/72 (70.8)
Visual impairment (n/N, %)	112/112 (100)
Insulin resistance (n/N, %)	47/80 (58.8)
Type 2 diabetes mellitus (n/N, %)	25/66(37.9)
Cardiac abnormalities (n/N, %)	46/79 (58.2)
Hepatic steatosis (n/N, %)	40/60 (66.7)
Elevated ALT/AST (n/N, %)	27/65 (41.5)

Note: ALT: Alanine Aminotransferase; AST: Aspartate Aminotransferase

### Spectrum of *ALMS1* variants in Chinese patients

**Fig. 2:**
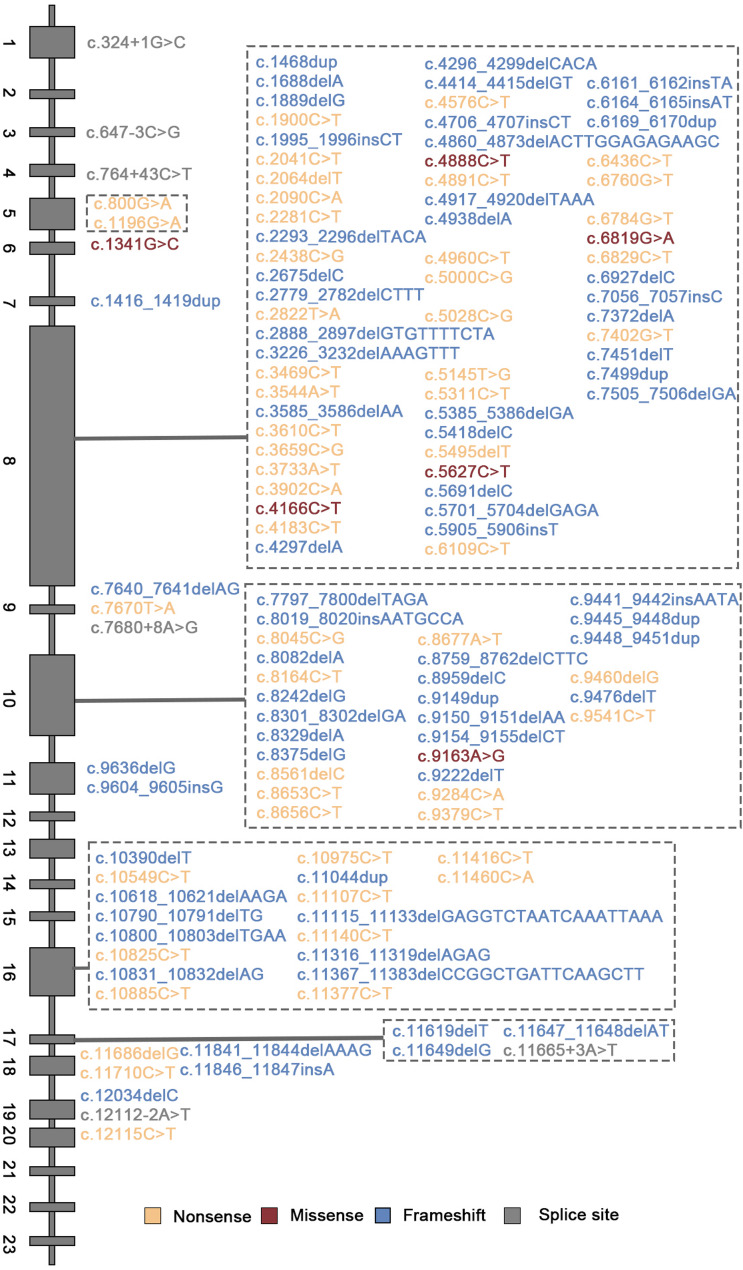
Pathogenic Variant Spectrum of the *ALMS1* Gene. Each black square on the horizontal axis represents an exon. Yellow squares denote nonsense mutations, red squares indicate missense mutations, blue squares represent frameshift mutations, and grey squares signify in-frame mutations. The figure illustrates the distribution of pathogenic variants in the *ALMS1* gene

**Fig. 3:**
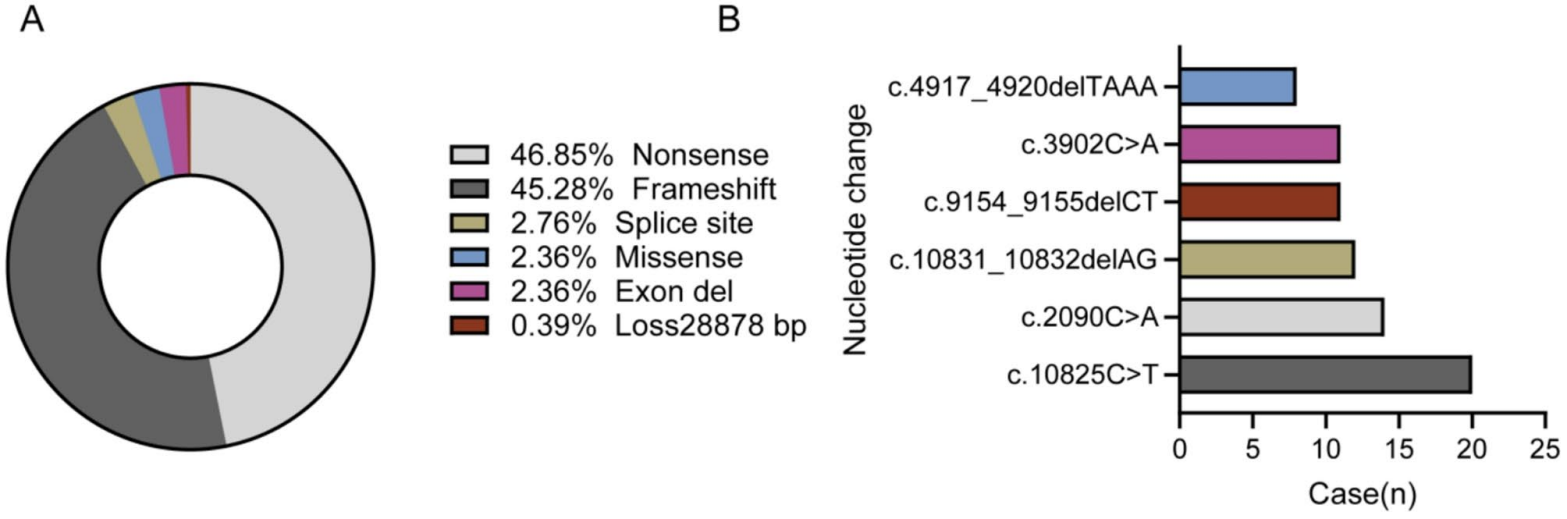
Basic Features of the Chinese Cohort (**A**). Variant types in 127 ALMS patients. (**B**) Mutation hotspots in the ALMS gene cohort

WES identified 132 distinct *ALMS1* variants among 127 patients (a total of 254 variants; Fig. 2), including nonsense mutations (46.85%, 119/254), frameshift mutations (45.28%, 115/254), splice-site mutations (2.76%, 7/254), missense mutations (2.36%, 6/254), exon deletions (2.36%, 6/254), and segmental gene deletions (0.39%, 1/254) (Fig. 3A). All patients carried biallelic P/LP variants, consistent with autosomal recessive inheritance. Among these, 64 variants were novel, being reported for the first time internationally. These novel variants were distributed across exons 4, 6, 8, 9, 10, 11, and 16–19 (Table 2), all with an allele frequency below 0.1%. Notably, a c.1341G > C missense variant was identified for the first time in exon 6 (Table 2).

**Table 2:** Novel variants in *ALMS1* identified in this study

Number	Exon	Nucleotide change	Amino acid change	Variant Type	Source	ACMG
1	4	c.764 + 43 C > T	p.?	Splice site	NA	LP
2	6	c.1341G > C	p.Lys447Asn	Missense	F	LP
3	8	c.1688delA	p.Asp563Alafs*33	Frameshift	F	LP
4	8	c.1995_1996insCT	p.Ser666Leufs*9	Frameshift	F	P
5	8	c.2041 C > T	p.Arg681Ter	Nonsense	M	P
6	8	c.2064delT	p.Leu689Ter	Nonsense	F	P
7	8	c.2296_2299delTCAC	p.Ser766Lysfs*13	Frameshift	M	P
8	8	c.2438 C > G	p.Ser813Ter	Nonsense	NA	LP
9	8	c.2675delC	p.Pro892Leufs*42	Frameshift	M	LP
10	8	c.2888_2897delGTGTTTTCTA	p.Ser963Thrfs*15	Frameshift	M	LP
11	8	c.3226_3232delAAAGTTT	p.Lys1076Glnfs*10	Frameshift	M	P
12	8	c.3469 C > T	p.Gln1157Ter	Nonsense	M	P
13	8	c.3610 C > T	p.Gln1204Ter	Nonsense	M	P
14	8	c.3659 C > G	p.Ser1220Ter	Nonsense	F	P
15	8	c.4297delA	p.Thr1433Glnfs*40	Frameshift	M	LP
16	8	c.4296_4299delCACA	p.His1432Glnfs*40	Frameshift	F	P
17	8	c.4576 C > T	p.Gln1526Ter	Nonsense	F	P
18	8	c.4706_4707delinsCTCT	p.Thr1570Leufs*28	Frameshift	NA	LP
19	8	c.4860_4873delACTTGGAGAGAAGC	p.Leu1621Hisfs*15	Frameshift	F	LP
20	8	c.5385_5386delGA	p.Lys1796Aspfs*30	Frameshift	F	P
21	8	c.5495delT	p.Leu1832Ter	Nonsense	M	P
22	8	c.5691delC	p.Ser1898Glnfs*22	Frameshift	NA	LP
23	8	c.6109 C > T	p.Gln2037Ter	Nonsense	M	P
24	8	c.6161_6162insTA	p.Lys2054Asnfs*21	Frameshift	M	LP
25	8	c.6164_6165insAT	p.Asn2056Serfs*19	Frameshift	F	P
26	8	c.6760G > T	p.Glu2254Ter	Nonsense	NA	P
27	8	c.6784G > T	p.Glu2262Ter	Nonsense	M	P
28	8	c.7056_7057insC	p.Glu2353Argfs*4	Frameshift	M	LP
29	8	c.7451delT	p.Val2484Glyfs*11	Frameshift	M	LP
30	8	c.7499delinsTT	p.Leu2501Thrfs*25	Frameshift	NA	LP
31	8	c.7505_7506delGA	p.Arg2502Lysfs*23	Frameshift	NA	LP
32	9	c.7670T > A	p.Leu2557Ter	Nonsense	F	P
33	9	c.7680 + 8 A > G	p.?	Splice site	M	P
34	10	c.7797_7800delTAGA	p.Arg2600Thrfs*13	Frameshift	M	LP
35	10	c.8082delA	p.Val2695Trpfs*15	Frameshift	F	P
36	10	c.8242delG	p.Val2748Trpfs*39	Frameshift	M	P
37	10	c.8301_8302delGA	p.Lys2768Asnfs*8	Frameshift	M	LP
38	10	c.8329delA	p.Met2777Cysfs*10	Frameshift	NA	P
39	10	c.8561delC	p.Leu2855Ter	Nonsense	F	LP
40	10	c.8653 C > T	p.Gln2885Ter	Nonsense	F	P
41	10	c.8959delC	p.Pro2987Profs*9	Frameshift	F	LP
42	10	c.9149delinsTT	p.Thr3051Asnfs*12	Frameshift	M	P
43	10	c.9150_9151delAA	p.Thr3051Serfs*11	Frameshift	F	P
44	10	c.9222delT	p.Ala3075Leufs*6	Frameshift	F	LP
45	10	c.9284 C > A	p.Ser3095Ter	Nonsense	NA	P
46	10	c.9445_9448delinsCAGACAGA	p.Asn3150Thrfs*3	Frameshift	F	P
47	10	c.9448_9451delinsAATAAATA	p.Ser3151Lysfs*2	Frameshift	M	LP
48	10	c.9460delG	p.Val3154Ter	Nonsense	NA	P
49	11	c.9636delG	p.Thr3213Profs*19	Frameshift	F	P
50	11	c.9604delinsGG	p.Val3202Glyfs*17	Frameshift	F	P
51	16	c.10390delT	p.Ser3464Profs*45	Frameshift	F	LP
52	16	c.10618_10621delAAGA	p.Lys3540Glnfs*6	Frameshift	F	P
53	16	c.10800_10803delTGAA	p.Glu3601Cysfs*60	Frameshift	F	LP
54	16	c.11044delinsAA	p.Ser3682Lysfs*7	Frameshift	M	P
55	16	c.11,140 C > T	p.Gln3714Ter	Nonsense	F	P
56	16	c.11367_11383delCCGGCTGATTCAAGCTT	p.Arg3790Trpfs*3	Frameshift	M	LP
57	16	c.11,377 C > T	p.Gln3793Ter	Nonsense	De novo	P
58	16	c.11,460 C > A	p.Tyr3820Ter	Nonsense	M	LP
59	17	c.11619delT	p.Thr3874Leufs*11	Frameshift	M	LP
60	17	c.11649delG	p.Met3883Ilefs*2	Frameshift	F	LP
61	18	c.11686delG	p.Val3896Ter	Nonsense	F	P
62	18	c.11841_11844delAAAG	p.Arg3947Serfs*2	Frameshift	F	P
63	18	c.11846_11847insA	p.Asn3952Leufs*10	Frameshift	F	P
64	19	c.12034delC	p.Leu4012Trpfs*19	Frameshift	F	P

Note: M: Mother; F: Father; P: Pathogenic; LP: Likely Pathogenic; NA: Not Available

The hotspot variant c.10,825 C > T was predominant, accounting for 7.87% (20/254) of the total, followed by c.2090 C > A (5.51%, 14/254) and c.10831_10832delAG (4.72%, 12/254) (Fig. 3B). Mutation hotspots were primarily located in exons 8, 10, and 16, contributing to 44.88%, 19.29%, and 24.02% of all variants, respectively. It is noteworthy that although exon 16 accounts for only 3.5% of the *ALMS1* coding sequence, it harbored 24.02% of pathogenic variants. Additionally, in 108 patients who completed parental verification, only 1.85%(4/216) of variants were identified as de novo. Three patients had one de novo allele maternally inherited on the other chromosome, and one had a paternal inherited variant. Affected siblings were observed in 10 families, underscoring the necessity of early genetic diagnosis for reproductive planning.

### Cardiac evaluations

**Fig. 4:**
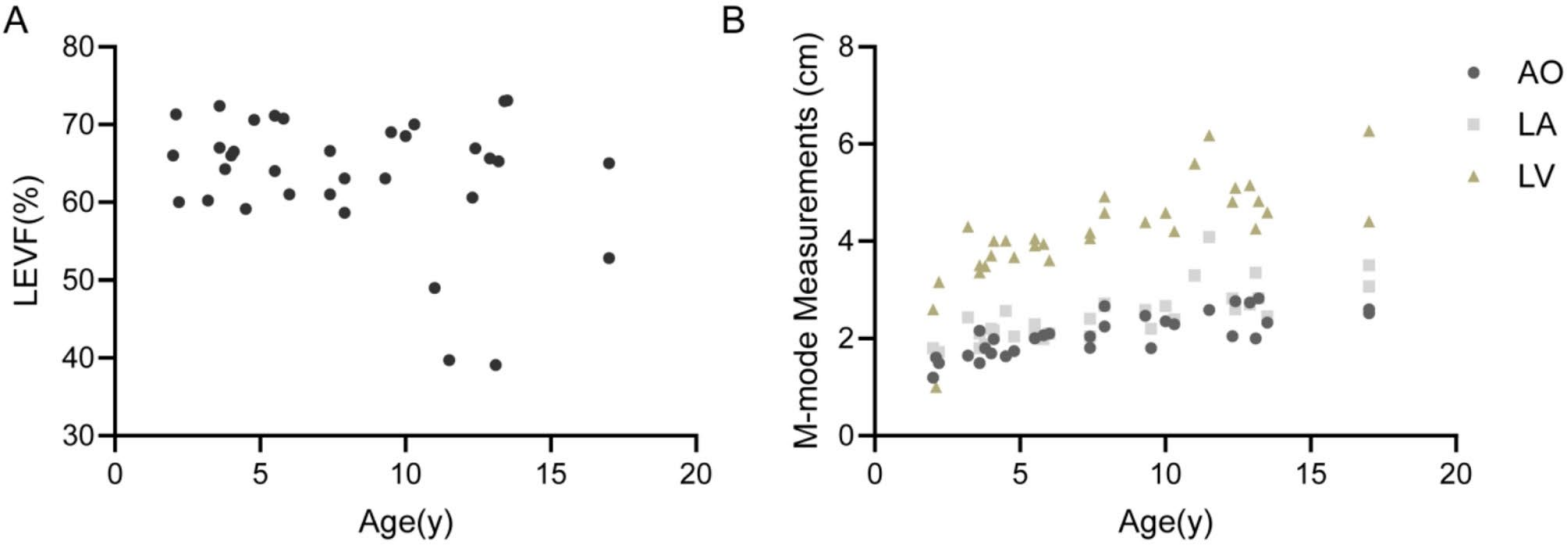
Cardiac Examination Findings in LVEF, AO, LA and LV with 34 ALMS Patients. (**A**) Distribution of LVEF in 34 ALMS patients with available echocardiographic data. (**B**) Scatter plots showing the relationship between age and cardiac chamber dimensions, including AO, LA and LV

More than half of the patients (58.2%, 46/79) exhibited cardiac abnormalities. The study included 34 genetically confirmed ALMS patients who underwent echocardiographic evaluation (Fig. 4), comprising 20 males (58.8%) with a median age of 7.65 years (range: 2.1–13.4 years) and 14 females (41.2%) with a median age of 6.95 years (range: 2.0–17.0 years). Clinical records revealed infantile-onset cardiomyopathy in 11 patients (32.4%), and left atrial and/or left ventricular enlargement in 24 patients (70.6%).

Regarding cardiac function, the mean LVEF was 63.54% ± 8.20%, (range: 39.11%-73.11%). The lowest LVEF was 39.1% in a 13.1-year-old boy, and the highest LVEF was 73.1% in a 13.5-year-old girl. Three patients demonstrated a reduced LVEF (< 50%). Although the majority of patients maintained LVEF within the normal range, the distribution was scattered, with notable inter-individual variability increasing with age (Fig. 4A).

Mild mitral or tricuspid regurgitation (MR/TR) was observed in 32 patients (94.1%), including 3 with moderate regurgitation. The dimensions of the aortic root (AO), left atrium (LA), and left ventricle (LV) progressively increased with age, with LV enlargement being the most prominent (Fig. 4B), further supporting that cardiomyopathy in ALMS patients predominantly manifests as progressive dilated cardiomyopathy.

### Retrospective analysis of infantile cardiomyopathy

This study conducted a retrospective analysis of 11 patients with a history of infantile cardiomyopathy. The mean age at onset was 0.34 ± 0.31 years, the mean age at diagnosis was 1.83 ± 2.59 years, and the current mean age was 7.55 ± 3.93 years. The male-to-female ratio was 7:4 (Table 3). No confirmed deaths due to infantile cardiomyopathy were identified among the study cohort. Apart from cardiac abnormalities, the clinical profiles of these patients were comparable to the overall cohort included in this study (Tables 1 and 3), including visual impairment (100%), obesity (90.9%, 10/11), hearing loss (62.5%, 5/8), insulin resistance (55.6%, 5/9) and type 2 diabetes mellitus (44.4%, 4/9). Liver involvement was also frequent (steatosis 54.5%, 6/11; elevated ALT/AST 45.5%, 5/11). These findings highlight that ALMS may manifest with both cardiac and sensory impairment in infancy, underscoring the need for heightened clinical vigilance for early symptom recognition and comprehensive assessment to facilitate timely diagnosis and intervention.

**Table 3:** Infantile-Onset cardiomyopathy features in 11 ALMS cases

Features	Frequency (*n*/*N*, %)
Age at onset of initial symptoms (mean ± SD, y)	0.34 ± 0.31
Age at diagnosis (mean ± SD, y)	1.83 ± 2.59
Current age (mean ± SD, y)	7.55 ± 3.93
Gender (M/F)	7/4
Obesity (n/N, %)	10/11 (90.9)
Hearing impairment (n/N, %)	5/8 (62.5)
Visual impairment (n/N, %)	11/11 (100)
Insulin resistance (n/N, %)	5/9 (55.6)
Type 2 diabetes mellitus (n/N, %)	4/9 (44.4)
Cardiac abnormalities (n/N, %)	11/11 (100)
Hepatic steatosis (n/N, %)	6/11 (54.5)
Elevated ALT/AST (n/N, %)	5/11 (45.5)

Note: ALT: Alanine Aminotransferase; AST: Aspartate Aminotransferase

**Fig. 5:**
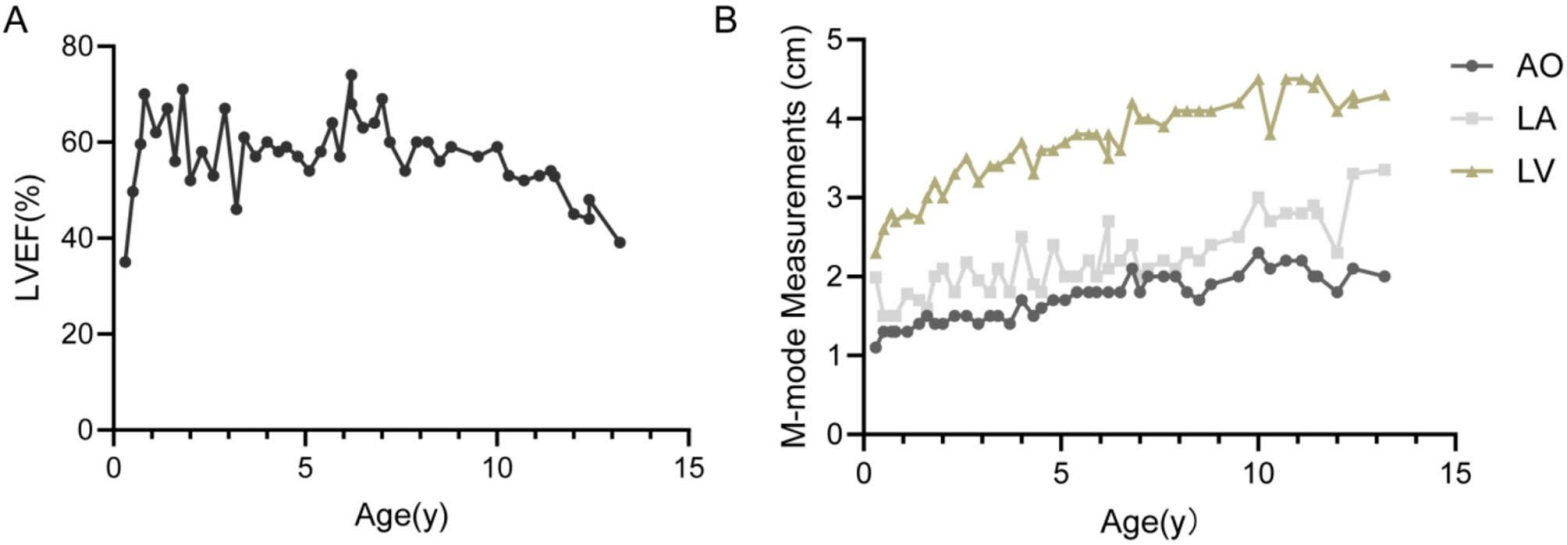
Longitudinal Changes in LVEF, AO, LA and LV During Follow-up in a Pediatric Patient with ALMS. (**A**) Longitudinal Changes in LVEF. (**B**) Longitudinal Changes in AO, LA and LV

In one representative patient with a history of infantile cardiomyopathy who underwent serial echocardiographic evaluations every 3–6 months over a 13-year follow-up period, the study constructed a longitudinal profile (Fig. 5). After initial treatment for reduced LVEF during infancy, the patient’s LVEF remained largely above 55% under regular follow-up and symptomatic management but showed a gradual decline during adolescence, with the most recent LVEF measured at 39.73% (Fig. 5A). Throughout the follow-up, the patient consistently exhibited left atrial and ventricular enlargement, mild mitral and tricuspid regurgitation, and endocardial thickening. Progressive increases in the diameters of the AO, LA, and LV were observed, with LV enlargement being particularly prominent (Fig. 5B), suggesting a progressive structural remodeling of the heart. Additionally, routine electrocardiographic monitoring revealed two episodes of sinus arrhythmia and six episodes of sinus tachycardia during the follow-up period (Supplementary Table S1).

### Genotype-phenotype correlation of cardiac abnormalities

Among the 34 patients who underwent echocardiographic evaluation, this study revealed that those with infantile-onset cardiac disease (*n* = 11) more frequently harbored variants in exons 8 and 16, suggesting a potential genotype-phenotype correlation. Therefore, this study aimed to investigate genotype-phenotype associations in ALMS patients.

This study compared patients with a history of infantile-onset cardiomyopathy to those without such a history. Overall, 72.7% (8/11) of patients with infantile-onset cardiac disease carried at least one truncating variant in exon 16, compared to only 30.4% (7/23) of those without a history of cardiomyopathy (Table 4), and this difference was statistically significant according to Pearson’s chi-square test (*p* < 0.05). However, Fisher’s exact test for variants in exon 8 did not reveal a statistically significant difference. Additional analysis found no significant enrichment of exon 16 variants among patients with type 2 diabetes (*p* >0.05), suggesting that these variants may not indicate overall disease severity within this cohort. This finding differs to some extent from the results reported by Marshall et al. [24], who proposed that exon 16 variants might be associated with more severe systemic manifestations in ALMS. These findings suggest that the region encoded by exon 16 may be critical for maintaining cardiac function. Nonetheless, due to the limited sample size, these observations warrant further validation in larger cohorts.

**Table 4:** Genotype-Phenotype correlation for Infantile-onset cardiomyopathy ALMS patient

Subjects	Patients with a history of infantile-onset cardiomyopathy, *N* = 11	Patients without a history of infantile-onset cardiomyopathy, *N* = 23	*p* Value
Age (mean ± SD, y)	5.85 ± 3.46	9.27 ± 4.38	0.03
Gender (male/female)	7/4	13/10	0.69
Carrying variants with exon 8, n (%)	9(81.8)	17(73.9)	1.00
Carrying variants with exon 16, n (%)	8(72.7)	7(30.4)	0.02

## Discussion

This study established the largest clinical and genetic cohort of ALMS patients in China to date, systematically characterizing the clinical features and mutation spectrum of the *ALMS1* gene in 127 confirmed individuals. Notably, this study identified 64 novel P/LP mutations, representing the first report of these variants worldwide, thereby significantly enriching the global mutation database for this rare disorder.

At the genetic level, *ALMS1* mutations in this cohort were predominantly nonsense (46.85%) and frameshift variants (45.28%), with mutational hotspots concentrated in exons 8, 10, and 16. These findings are largely consistent with previous reports based on the HGMD database and studies in European cohorts such as Polish and Turkey populations [15–17]. However, this study also observed several high-frequency, population-specific variants in the Chinese cohort [2, 18], such as c.2090 C >A (5.51%, 14/254), suggesting a potential ethnic-specific mutation spectrum that merits further population genetics studies and functional validation.

Clinically, visual impairment, obesity, hearing loss, hepatic steatosis, and cardiac abnormalities were the most prevalent manifestations, consistent with the classical phenotypic profile of ALMS [4, 19]. Among evaluable patients, insulin resistance was confirmed in 58.8%, and those with insulin resistance were significantly older (*P* < 0.001), demonstrating an age-related progression of metabolic dysfunction. Although ALMS has traditionally been classified as a ciliopathy, accumulating evidence suggests that ALMS1 also exerts important non-ciliary functions in intracellular trafficking, cytoskeletal organization, and metabolic regulation [9–11]. Recent studies have expanded that hematopoietic stem cell progenitors can drive systemic metabolic disturbances in ALMS, highlighting the contribution of metabolic and immune dysregulation to the disorder’s multisystem pathophysiology [25, 26].

Notably, the detection rate of cardiac abnormalities in our cohort (58.2%) was significantly higher than that reported in previous studies [15, 17, 27]. This finding suggests that cardiac involvement may be more prominent in Chinese patients, potentially due to earlier cardiac screening, genetic background differences, or environmental factors such as diet and physical activity. Furthermore, the incidence of infantile-onset cardiomyopathy reached 32.4% (11/34), underscoring the critical importance of cardiac monitoring in the early diagnosis and management of ALMS. Importantly, no fatal outcomes were recorded during follow-up, indicating that early recognition and appropriate management can stabilize cardiac function [28].

A major innovation of this study was the retrospective and longitudinal analysis of clinical phenotypes in 11 patients with infantile-onset cardiomyopathy. This analysis revealed that most patients showed improvement in LVEF following intervention, providing preliminary evidence for stage-dependent functional reversibility in ALMS-associated cardiomyopathy. Additionally, this study report for the first time the long-term echocardiographic follow-up of an infantile-onset cardiomyopathy patient, highlighting that clinical management should avoid overly pessimistic expectations and instead focus on early diagnosis and targeted treatment. This finding fills a critical gap in understanding the progression of ALMS-associated cardiomyopathy and provides a valuable foundation for future mechanistic research and interventional trials.

Regarding treatment, there are currently no disease-specific pharmacologic therapies proven to be effective for ALMS-associated cardiomyopathy [1, 29]. Management in our cohort, particularly among patients with infantile-onset cardiomyopathy, remained largely supportive and symptom-directed. Standard pediatric heart failure regimens were employed in these infants and young children, including angiotensin-converting enzyme (ACE) inhibitors or angiotensin receptor blockers (ARBs), β-blockers, and mineralocorticoid receptor antagonists, with regular monitoring of blood pressure, cardiac function, and electrolyte balance. In cases presenting with significantly reduced contractility or overt heart failure, short courses of digoxin were administered under close clinical supervision. For concomitant metabolic or hepatic abnormalities, hepatoprotective agents such as glycyrrhizin compound and coenzyme Q10 were used in selected cases, while lifestyle modification and metformin were prescribed for patients with insulin resistance or type 2 diabetes, following pediatric endocrinology recommendations. Collectively, these findings suggest that early recognition and standard heart-failure management can lead to functional stabilization and improved survival in infantile-onset cases, although no ALMS1-targeted therapy has yet been established.

In terms of genotype-phenotype correlation, although no direct association between specific pathogenic variants and cardiomyopathy risk was identified, this study observed that truncating variants in exon 16 were significantly enriched among patients with infantile-onset cardiomyopathy (72.7% vs. 30.4%, *p* < 0.05). Given that exon 16 encodes a region involved in centrosomal docking and ciliary elongation, structural defects in this domain could theoretically impair cardiomyocyte mechanotransduction, leading to early ventricular remodeling [24, 30]. This finding provides important clues for further genotype-phenotype investigations in ALMS. Notably, variants in exon 8 did not show a statistically significant difference, which may imply a domain-specific effect on organ development, highlighting the particular importance of the exon 16 coding region in cardiac development. Despite this statistical association, these results should be interpreted cautiously given the modest sample size and lack of functional validation. Future in vitro and animal model studies focusing on exon 16 truncations are essential to clarify their pathogenic mechanisms.

This study also has certain limitations. First, as ALMS remains a rare disease in China, the time span of diagnosis and treatment among patients was considerable, and some clinical data and imaging follow-up records were incomplete, potentially leading to biases in phenotypic frequency assessments. Additionally, constrained by various factors, this study did not systematically perform functional analyses of novel variants, nor did it integrate multi-omics approaches such as transcriptomics or proteomics, thus limiting the mechanistic interpretation of how *ALMS1* mutations lead to multisystem dysfunction. In addition, early childhood data such as the age of onset of nystagmus or infantile cardiomyopathy partly relied on parental reporting, possibly leading to underestimation of symptom onset. Finally, as most patients were pediatric, long-term outcomes in adulthood, especially regarding progressive cardiac and renal fibrosis, remain to be determined.

In summary, this study represents a critical step forward in delineating the clinical and genetic characteristics of ALMS patients in China. The establishment of a comprehensive clinical-genetic database not only facilitates early diagnosis, risk prediction, and follow-up management but also provides crucial support for advancing precision medicine in the field of rare diseases. Future research should include longitudinal follow-up studies to re-evaluate echocardiographic and multisystem changes in this cohort, thereby enabling a better understanding of disease progression over time. Besides, integration of animal models, cellular functional assays, and multi-omics analyses will be essential to further elucidate the pathogenic mechanisms of *ALMS1* variants, particularly their roles in cardiomyopathy, obesity, and hepatic injury, and to explore potential therapeutic targets and individualized treatment strategies.

## Supplementary Information

Below is the link to the electronic supplementary material.

Supplementary Material 1

## Data Availability

The data that support the findings of this study are available from the corresponding authors upon reasonable request.

## References

[1] Tahani N, Maffei P, Dollfus H, Paisey R, Valverde D, Milan G, et al. Consensus clinical management guidelines for Alström syndrome. Orphanet J Rare Dis. 2020;15:253. 10.1186/s13023-020-01468-8. 32958032 10.1186/s13023-020-01468-8 PMC7504843

[2] Bea-Mascato B, Valverde D. Genotype-phenotype associations in Alström syndrome: a systematic review and meta-analysis. J Med Genet. 2023;61:18–26. 10.1136/jmg-2023-109175. 37321834 10.1136/jmg-2023-109175 PMC10803979

[3] Bond J, Flintoff K, Higgins J, Scott S, Bennet C, Parsons J, et al. The importance of seeking ALMS1 mutations in infants with dilated cardiomyopathy. J Med Genet. 2005;42:e10. 10.1136/jmg.2004.026617. 15689433 10.1136/jmg.2004.026617 PMC1735981

[4] Marshall JD, Beck S, Maffei P, Naggert JK. Alström syndrome. Eur J Hum Genet EJHG. 2007;15:1193–202. 10.1038/sj.ejhg.5201933. 17940554 10.1038/sj.ejhg.5201933

[5] Marshall JD, Maffei P, Collin GB, Naggert JK. Alström syndrome: genetics and clinical overview. Curr Genomics. 2011;12:225–35. 10.2174/138920211795677912. 22043170 10.2174/138920211795677912 PMC3137007

[6] Badano JL, Mitsuma N, Beales PL, Katsanis N. The ciliopathies: an emerging class of human genetic disorders. Annu Rev Genomics Hum Genet. 2006;7:125–48. 10.1146/annurev.genom.7.080505.115610. 16722803 10.1146/annurev.genom.7.080505.115610

[7] Andersen JS, Wilkinson CJ, Mayor T, Mortensen P, Nigg EA, Mann M. Proteomic characterization of the human centrosome by protein correlation profiling. Nature. 2003;426:570–4. 10.1038/nature02166. 14654843 10.1038/nature02166

[8] Hearn T. ALMS1 and Alström syndrome: a recessive form of metabolic, neurosensory and cardiac deficits. J Mol Med Berl Ger. 2019;97:1–17. 10.1007/s00109-018-1714-x. 10.1007/s00109-018-1714-x PMC6327082 30421101

[9] Collin GB, Cyr E, Bronson R, Marshall JD, Gifford EJ, Hicks W, et al. Alms1-disrupted mice recapitulate human Alström syndrome. Hum Mol Genet. 2005;14:2323–33. 10.1093/hmg/ddi235. 16000322 10.1093/hmg/ddi235 PMC2862911

[10] Girard D, Petrovsky N. Alström syndrome: insights into the pathogenesis of metabolic disorders. Nat Rev Endocrinol Nat Publishing Group. 2011;7:77–88. 10.1038/nrendo.2010.210. 10.1038/nrendo.2010.210 21135875

[11] Geberhiwot T, Baig S, Obringer C, Girard D, Dawson C, Manolopoulos K, et al. Relative adipose tissue failure in Alström syndrome drives Obesity-Induced insulin resistance. Diabetes. 2021;70:364–76. 10.2337/db20-0647. 32994277 10.2337/db20-0647 PMC7881858

[12] Bea-Mascato B, Gómez-Castañeda E, Sánchez-Corrales YE, Castellano S, Valverde D. Loss of the centrosomal protein ALMS1 alters lipid metabolism and the regulation of extracellular matrix-related processes. Biol Direct. 2023;18:84. 10.1186/s13062-023-00441-2. 38062477 10.1186/s13062-023-00441-2 PMC10704752

[13] Álvarez-Satta M, Lago-Docampo M, Bea-Mascato B, Solarat C, Castro-Sánchez S, Christensen ST, et al. ALMS1 regulates TGF-β signaling and morphology of primary cilia. Front Cell Dev Biol. 2021;9:623829. 10.3389/fcell.2021.623829. 33598462 10.3389/fcell.2021.623829 PMC7882606

[14] Marshall JD, Muller J, Collin GB, Milan G, Kingsmore SF, Dinwiddie D, et al. Alström syndrome: mutation spectrum of ALMS1. Hum Mutat. 2015;36:660–8. 10.1002/humu.22796. 25846608 10.1002/humu.22796 PMC4475486

[15] Brofferio A, Sachdev V, Hannoush H, Marshall JD, Naggert JK, Sidenko S, et al. Characteristics of cardiomyopathy in Alström syndrome: prospective single-center data on 38 patients. Mol Genet Metab. 2017;121:336–43. 10.1016/j.ymgme.2017.05.017. 28610912 10.1016/j.ymgme.2017.05.017 PMC5555226

[16] Zmyslowska A, Borowiec M, Antosik K, Ploski R, Ciechanowska M, Iwaniszewska B, et al. Genetic evaluation of patients with Alström syndrome in the Polish population. Clin Genet. 2016;89:448–53. 10.1111/cge.12656. 26283575 10.1111/cge.12656

[17] Ozantürk A, Marshall JD, Collin GB, Düzenli S, Marshall RP, Candan Ş, et al. The phenotypic and molecular genetic spectrum of Alström syndrome in 44 Turkish kindreds and a literature review of Alström syndrome in Turkey. J Hum Genet. 2015;60:1–9. 10.1038/jhg.2014.85. 25296579 10.1038/jhg.2014.85 PMC5460767

[18] Zhang Q, Ding Y, Feng B, Tang Y, Chen Y, Wang Y, et al. Molecular and phenotypic expansion of Alström syndrome in Chinese patients. Front Genet. 2022;13:808919. 10.3389/fgene.2022.808919. 35211159 10.3389/fgene.2022.808919 PMC8861322

[19] Paisey RB, Steeds R, Barrett T, Williams D, Geberhiwot T, Gunay-Aygun M. Alström syndrome. In: Adam MP, Feldman J, Mirzaa GM, Pagon RA, Wallace SE, Amemiya A, editors. GeneReviews^®^. Seattle (WA). University of Washington, Seattle; 1993. http://www.ncbi.nlm.nih.gov/books/NBK1267/. 20301444

[20] Hanson H, Astiazaran-Symonds E, Amendola LM, Balmaña J, Foulkes WD, James P, et al. Management of individuals with germline pathogenic/likely pathogenic variants in CHEK2: A clinical practice resource of the American college of medical genetics and genomics (ACMG). Genet Med Off J Am Coll Med Genet. 2023;25:100870. 10.1016/j.gim.2023.100870. 10.1016/j.gim.2023.100870 PMC10623578 37490054

[21] Keskin M, Kurtoglu S, Kendirci M, Atabek ME, Yazici C. Homeostasis model assessment is more reliable than the fasting glucose/insulin ratio and quantitative insulin sensitivity check index for assessing insulin resistance among obese children and adolescents. Pediatrics. 2005;115:e500–503. 10.1542/peds.2004-1921. 15741351 10.1542/peds.2004-1921

[22] Lang RM, Badano LP, Mor-Avi V, Afilalo J, Armstrong A, Ernande L, et al. Recommendations for cardiac chamber quantification by echocardiography in adults: an update from the American society of echocardiography and the European association of cardiovascular imaging. J Am Soc Echocardiogr Off Publ Am Soc Echocardiogr. 2015;28:1–e3914. 10.1016/j.echo.2014.10.003. 10.1016/j.echo.2014.10.003 25559473

[23] Lopez L, Saurers DL, Barker PCA, Cohen MS, Colan SD, Dwyer J, et al. Guidelines for performing a comprehensive pediatric transthoracic echocardiogram: recommendations from the American society of echocardiography. J Am Soc Echocardiogr Off Publ Am Soc Echocardiogr. 2024;37:119–70. 10.1016/j.echo.2023.11.015. 10.1016/j.echo.2023.11.015 38309834

[24] Marshall JD, Hinman EG, Collin GB, Beck S, Cerqueira R, Maffei P, et al. Spectrum of ALMS1 variants and evaluation of genotype-phenotype correlations in Alström syndrome. Hum mutat. Volume 28. John Wiley & Sons, Ltd; 2007. pp. 1114–23. 10.1002/humu.20577. 10.1002/humu.20577 17594715

[25] Dassie F, Albiero M, Bettini S, Cappellari R, Milan G, Ciciliot S, et al. Hematopoietic stem cells and metabolic deterioration in Alström syndrome, a rare genetic model of the metabolic syndrome. Endocrinology. 2023;164:bqad011. 10.1210/endocr/bqad011. 36702623 10.1210/endocr/bqad011

[26] Ademolu AB, Geberhiwot T. Hematopoietic stem cell progenitors driving metabolic disturbance in Alström syndrome. 10.1210/endocr/bqad110. Accessed 12 Oct 2025. 10.1210/endocr/bqad110 37449512

[27] Marshall JD, Bronson RT, Collin GB, Nordstrom AD, Maffei P, Paisey RB, et al. New Alström syndrome phenotypes based on the evaluation of 182 cases. Arch Intern Med. 2005;165:675–83. 10.1001/archinte.165.6.675. 15795345 10.1001/archinte.165.6.675

[28] Van Huffel J, Derycke E, Detaille T, Moniotte S, Hubrechts J. Infantile dilated cardiomyopathy in Alström. Syndrome Cureus 16:e74895. 10.7759/cureus.74895 10.7759/cureus.74895 PMC11686418 39742192

[29] Dassie F, Favaretto F, Bettini S, Parolin M, Valenti M, Reschke F, et al. Alström syndrome: an ultra-rare Monogenic disorder as a model for insulin resistance, type 2 diabetes mellitus and obesity. Endocrine. 2021;71:618–25. 10.1007/s12020-021-02643-y. 33566311 10.1007/s12020-021-02643-y

[30] Jagger D, Collin G, Kelly J, Towers E, Nevill G, Longo-Guess C, et al. Alström syndrome protein ALMS1 localizes to basal bodies of cochlear hair cells and regulates cilium-dependent planar cell Polarity. Hum Mol Genet. 2011;20:466–81. 10.1093/hmg/ddq493. 21071598 10.1093/hmg/ddq493 PMC3016908

