# Supplementary material for: Alström syndrome: a cross-sectional and follow-up study of 127 patients in China, highlighting genetic variant spectrum and cardiac features
Source: Orphanet J Rare Dis. 2025 Dec 29;20:630. doi: 10.1186/s13023-025-04139-8 (PMC12751321; doi:10.1186/s13023-025-04139-8)
Supplement: Supplementary file 1 — Supplementary Material 1 [file 13023_2025_4139_MOESM1_ESM.docx]

| **Supplementary Table S1. Longitudinal Changes in LVEF, AO, LA, LV, heart rhythm and heart rate During Follow-up in a Pediatric Patient with ALMS** | | | | | | |
| --- | --- | --- | --- | --- | --- | --- |
| Age (years) | LVEF (%) | AO (cm) | LA (cm) | LV (cm) | Heart Rhythm | Heart Rate |
| 0.3 | 35% | 1.1 | 1.99 | 2.3 | / | / |
| 0.5 | 49.70% | 1.3 | 1.5 | 2.6 | / | / |
| 0.7 | 59.60% | 1.3 | 1.5 | 2.8 | Sinus Rhythm | 115 |
| 0.8 | 70% | 1.3 | 1.5 | 2.7 | Sinus Tachycardia | 166 |
| 1.1 | 62% | 1.3 | 1.78 | 2.8 | Sinus Rhythm | 94 |
| 1.4 | 67% | 1.4 | 1.7 | 2.74 | Sinus Rhythm | 100 |
| 1.6 | 56% | 1.5 | 1.6 | 3 | Sinus Rhythm | 94 |
| 1.8 | 71% | 1.4 | 2 | 3.2 | Sinus Rhythm | 100 |
| 2.0 | 52% | 1.4 | 2.1 | 3 | Sinus Rhythm | 94 |
| 2.3 | 58% | 1.5 | 1.8 | 3.3 | Sinus Rhythm | 94 |
| 2.6 | 53% | 1.5 | 2.18 | 3.5 | Sinus Rhythm | 100 |
| 2.9 | 67% | 1.4 | 1.95 | 3.2 | / | / |
| 3.2 | 46% | 1.5 | 1.8 | 3.4 | Sinus Rhythm | 110 |
| 3.4 | 61% | 1.5 | 2.1 | 3.4 | Sinus Rhythm | 102 |
| 3.7 | 57% | 1.4 | 1.8 | 3.5 | Sinus Tachycardia | 164 |
| 4.0 | 60% | 1.7 | 2.5 | 3.7 | Sinus Rhythm | 92 |
| 4.3 | 58% | 1.5 | 1.9 | 3.3 | Sinus Tachycardia | 150 |
| 4.5 | 59% | 1.6 | 1.8 | 3.6 | Sinus Rhythm | 107 |
| 4.8 | 57% | 1.7 | 2.4 | 3.6 | Sinus Tachycardia | 143 |
| 5.1 | 54% | 1.7 | 2 | 3.7 | Sinus Rhythm | 107 |
| 5.4 | 58% | 1.8 | 2 | 3.8 | Sinus Tachycardia | 136 |
| 5.7 | 64% | 1.8 | 2.2 | 3.8 | Sinus Tachycardia | 106 |
| 5.9 | 57% | 1.8 | 2 | 3.8 | Sinus Arrhythmia | 86 |
| 6.2 | 74% | 1.8 | 2.7 | 3.5 | / | / |
| 6.2 | 68% | 1.8 | 2.1 | 3.8 | / | / |
| 6.5 | 63% | 1.8 | 2.2 | 3.6 | / | / |
| 6.8 | 64% | 2.1 | 2.4 | 4.2 | / | / |
| 7.0 | 69% | 1.8 | 2 | 4 | Sinus Arrhythmia | 80 |
| 7.2 | 60% | 2 | 2.1 | 4 | Sinus Rhythm | 84 |
| 7.6 | 54% | 2 | 2.2 | 3.9 | Sinus Rhythm | 98 |
| 7.9 | 60% | 2 | 2.1 | 4.1 | Sinus Rhythm | 81 |
| 8.2 | 60% | 1.8 | 2.3 | 4.1 | Sinus Rhythm | 74 |
| 8.5 | 56% | 1.7 | 2.2 | 4.1 | Sinus Rhythm | 91 |
| 8.8 | 59% | 1.9 | 2.4 | 4.1 | Sinus Rhythm | 99 |
| 9.5 | 57% | 2 | 2.5 | 4.2 | Sinus Rhythm | 87 |
| 10.0 | 59% | 2.3 | 3 | 4.5 | Sinus Rhythm | 93 |
| 10.3 | 53% | 2.1 | 2.7 | 3.8 | Sinus Rhythm | 86 |
| 10.7 | 52% | 2.2 | 2.8 | 4.5 | Sinus Rhythm | 98 |
| 11.1 | 53% | 2.2 | 2.8 | 4.5 | Sinus Rhythm | 92 |
| 11.4 | 54% | 2 | 2.9 | 4.4 | Sinus Rhythm | 84 |
| 11.5 | 52.90% | 2 | 2.8 | 4.5 | Sinus Rhythm | 70 |
| 12.0 | 45% | 1.8 | 2.3 | 4.1 | Sinus Rhythm | 92 |
| 12.4 | 44% | 2.1 | 3.3 | 4.3 | Sinus Rhythm | 74 |
| 12.4 | 48% | 2.1 | 3.3 | 4.2 | Sinus Rhythm | 88 |
| 13.2 | 39.11% | 2 | 3.35 | 4.3 | / | / |
| Note: AO: Aortic Valve Opening; LA: Left Atrium; LVEF: Left Ventricular Ejection Fraction | | | | | | |
